# Draft Genome Sequence of the Plant Growth-Promoting Bacterium *Pseudomonas* sp. SCPG-7, Isolated from Saline Soil

**DOI:** 10.1128/genomeA.00702-17

**Published:** 2017-08-10

**Authors:** Yajie Han, Bin Dai, Ying Zhou, Zhansheng Wu, Bang-Ce Ye

**Affiliations:** a School of Chemistry and Chemical Engineering, Shihezi University, Shihezi, China; b Lab of Biosystems and Microanalysis, State Key Laboratory of Bioreactor Engineering, East China University of Science and Technology, Shanghai, China

## Abstract

*Pseudomonas* sp. SCPG-7 was isolated from saline soil. The strain can increase the germination rate of cotton seeds and promote the growth of cotton seedlings under salt stress conditions. The genome is 6,256,198 bp long, containing 5,672 predicted open reading frames.

## GENOME ANNOUNCEMENT

The draft genome sequence of *Pseudomonas* sp. SCPG-7 has been completed. It was isolated from saline soil in a cotton field in Xinjiang Province, China. The strain can increase the germination rate of cotton seeds and promote the growth of cotton seedlings under salt stress conditions.

Probiotics are considered to be living microorganisms which, when administered in adequate amounts, can confer a beneficial physiological effect on the host (1). Plant growth-promoting bacteria are soil bacteria that facilitate plant growth and are often found in association with plant roots (rhizospheric) and sometimes within plant tissues (endophytic) (2, 3).

Pure cultures of *Pseudomonas* sp. SCPG-7 were confirmed on nutrient agar medium consisting of 10 g/liter of tryptone, 5 g/liter of beef extract, and 5 g/liter of NaCl at 30°C for 48 h. Analysis of the 16S rRNA gene sequences from single colonies allowed the assignment of the isolate to the genus *Pseudomonas*, with the highest sequence identity (99%) observed with the 16S rDNA gene of *Pseudomonas koreensis*. Members of the genus *Pseudomonas* have been identified as Gram-negative, opaque, ivory-colored bacteria that have irregular margins and are motile with a polar flagellum (4, 5).

The genome of strain SCPG-7 was determined by the Majorbio Company (Shanghai, China) using Illumina paired-end sequencing technology. Approximately 2,874,684 × 2 clean-data paired-end reads with a 94.15% Q20 value were generated. Read assembly was performed *de novo* using SOAPdenovo version 2.04 (http://soap.genomics.org.cn) (6).

The *de novo* assembly resulted in a 6,256,198-bp genome, with a G+C content of 60.07% and comprising 73 contigs. Scaffolding produced 60 supercontigs, of which the largest one (896,647 bp) represented 14.3% of the total assembly length. The genome was predicted using Glimmer version 3.02; the chromosome contains 2 copies of rRNAs, 61 tRNAs, and 5,672 protein-coding genes.

### Accession number(s).

The genome sequences have been deposited in NCBI GenBank under the accession number MVOO00000000.
